# Self-reported Ethnicity Changes over Time: A Population Level Study using Public Voter Databases

**DOI:** 10.23889/ijpds.v9i5.2591

**Published:** 2024-09-18

**Authors:** Sumayya Ziyad, Joseph Lam, Peter Christen, Rainer Schnell, Charini Nanayakkara

**Affiliations:** 1 Australian National University; 2 University College London; 3 University Duisburg-Essen

## Objectives

Administrative databases such as voter registries contain a wealth of information about a large proportion of a population. This is especially true when longitudinal information about individuals is available, such as changes in their names, addresses, or self-reported ethnic attributes. Apparent changes in ethnic attributes might be due to perceptions of majority evaluations of ethnic groups. In this project, we analysed snapshots of a publicly available US voter database from 2015 to 2022, exploring if and how recent presidential elections impacted changes in voters' self-reported ethnic attributes.

## Approach

Using bi-monthly snapshots of a publicly available US voter database from 2015 to 2022, we generated cohorts by linking active voters during seven twelve-month periods (2015/16 to 2021/22). These seven time periods included one mid-term and two presidential elections, and four control time spans before, between, and after these elections. We compared changes in self-reported ethnic attributes of voters during the times of elections versus the control time periods.

## Results

Preliminary results reveal a substantial change to self-reported ethnic attributes during the periods leading to an election. A majority of such changes are by individuals from a specific ethnic group choosing not to report an ethnicity, an indication of their preference to not disclose ethnicity-related sensitive information.

## Conclusions

We have shown how a publicly available voter database can be used to explore how self-reported ethnic attributes are modified by individual voters over time, and how the prevalence of such changes is influenced by political events such as presidential elections.

